# Repairing the Aged Parkinsonian Striatum: Lessons from the Lab and Clinic

**DOI:** 10.4172/2155-9899.1000476

**Published:** 2016-12-06

**Authors:** Natosha M Mercado, Timothy J Collier, Thomas Freeman, Kathy Steece-Collier

**Affiliations:** 1 Department of Translational Science & Molecular Medicine, College of Human Medicine, Michigan State University, Grand Rapids, MI 49503, USA; 2 Hauenstein Neuroscience Center, Mercy Health Saint Mary’s, Grand Rapids, Michigan 49503, USA; 3 Department of Neurosurgery and Brain Repair, Center of Excellence for Aging and Brain Repair, College of Medicine, University of South Florida, FL 33612, USA

## Abstract

The primary risk factor associated with Parkinson's disease (PD) is advanced age. While there are symptomatic therapies for PD, efficacy of these eventually wane and/or side-effects develop over time. An alternative experimental therapy that has received a great deal of attention over the past several decades has been neural transplantation aimed at replacing nigral dopamine (DA) neurons that degenerate in PD. However, in PD patients and parkinsonian rats, advanced age is associated with inferior benefit following intrastriatal grafting of embryonic DA neurons. Traditionally it has been thought that decreased therapeutic benefit results from the decreased survival of grafted DA neurons and the accompanying poor reinnervation observed in the aged host. However, recent clinical and preclinical data suggest that factors inherent to the aged striatum *per se* limit successful brain repair. In this short communication, we focus discussion on the implications of our recent grafting study in aged parkinsonian rats, with additional emphasis on a recent clinical report of the outcome of cell therapy in an aged PD patient with long-term (24 years) survival of DA neuron grafts. To address aging as a limiting factor in successful brain repair, we use the example of cell transplantation as a means to interrogate the environment of the aged striatum and identify factors that may, or may not, respond to interventions aimed at improving the prospects for adequate repair of the aged brain. We offer discussion of how these recent reports, in the context of other historical grafting studies, might provide new insight into specific risk factors that have potential to negatively impact all DA cell or terminal replacement strategies for clinical use in PD.

## Introduction

Parkinson’s disease (PD) is a relentlessly progressive neurodegenerative disorder impacting millions of people worldwide. There is currently no available treatment to slow disease progression and the benefit of best-medical treatment wanes and side effects develop over time. Thus, improving therapeutic options for individuals with PD is greatly needed. One experimental therapy that has received a great deal of attention for more than three decades is grafting of dopamine (DA) neurons to replace those lost in PD. While controversy surrounds the sources of the cells used for neural transplantation in PD [1–7], the rationale is strong and based on the premise that if a neurological syndrome can be linked to the loss of a specific population of neurons, then replacing these cellular elements should restore the lost function. While some PD patients have shown marked and lasting benefit following engraftment of primary DA neurons, many more have shown limited benefit and/or development of significant graft-derived side effects [1–4,6].

Aging is the primary risk factor for PD and the majority of patients that have received cell transplant therapy generally can be characterized as elderly. Yet, the impact of the environment of the aged brain on the response to cell therapy has received limited attention in laboratory studies and only now is being inferred from outcomes associated with PD patients transplanted many years previously. Decades of preclinical and clinical research on DA neuron grafting in PD have suggested three primary risk factors that are associated with graft failure: 1) advanced age of the host [8,2,9,10]; 2) extensive striatal DA denervation [1,4,5,11]; and most recently 3) alpha-synuclein (α-syn) pathology [12–16]. Arguably, all of these factors can be associated with the environment of the aging brain. Despite the importance of having identified such risk factors, it remains uncertain how these factors compromise graft success and thus accordingly, how to overcome them. In this short communication, we focus discussion on the implications of our recent grafting study in aged parkinsonian rats [8], with additional emphasis on a recent clinical report of the outcome of cell therapy in a PD patient receiving grafted cells 24 years prior to death at the age of 83 [17]. To address aging as a limiting factor in successful brain repair, we use the example of cell transplantation as a means to interrogate the environment of the aged striatum and identify factors that may, or may not, respond to interventions aimed at improving the prospects for adequate repair of the aged brain. We offer discussion of how these recent reports, in the context of other historical grafting studies, might provide new insight into the above-mentioned risk factors; factors that have potential to negatively impact all DA cell or terminal replacement strategies for clinical use.

## Identifying Potential Risk Factors for Cell Replacement Strategies in PD

### Aging and PD

The primary risk factor for PD is advanced age (e.g.: [18]). Advanced age also is a primary risk factor associated with inferior symptomatic benefit associated with intrastriatal grafts of embryonic DA neurons in both PD patients and parkinsonian rats [1,2,8,9]. Initial studies in aging parkinsonian rats grafted with the same number of cells as young animals attributed an observed reduction in graft-derived therapeutic benefit in aged rats to decreased survival of grafted DA neurons and accompanying poor reinnervation of the host striatum [9]. Indeed, extensive efforts have gone into identifying means of optimizing survival of grafted cells (reviewed in [8]). However, as discussed in Collier et al., [8] comparatively little effort has been devoted to understanding how the host environment impacts graft success outside of its influence on graft survival, per se, through altered immune and/or trophic support mechanisms. As evidenced from the reports of Li and colleagues [17] and Collier et al. [8], in PD patients and parkinsonian rats, respectively, the fact remains that even when obstacles that compromise graft survival are overcome, robust survival of grafted DA neurons does not necessarily equate to functional benefit. Understanding the disconnect between adequate survival of grafted neurons and predicted therapeutic benefit remains a critical issue in optimizing DA terminal replacement strategies as an alternative therapeutic option for PD.

### Impact of aging on graft success: Lessons from the laboratory

Within the aged, parkinsonian brain two primary risk factors, advanced age and DA depletion, combine to create an environment that significantly challenges the ability to re-establish new connections and meaningful circuitry between the new DA terminals and the host neurons. Indeed, the recent findings from Collier et al., [8] suggest that factors unique to aging appear to impede the establishment of synaptic re-wiring between grafted DA neurons and their principal striatal target, medium spiny neurons (MSNs). Briefly, in this study that was aimed at further elucidating the role of aging in inferior symptomatic benefit, we used a parkinsonian rat model to examine whether proportionally increasing the number of embryonic midbrain DA neurons grafted into the parkinsonian striatum of aged rats would translate into increased survival of transplanted DA cells, and consequently, enhanced behavioral recovery comparable to that achieved with neural grafting in younger parkinsonian rats.

We reasoned that since there is a proportional decrease in survival of grafted DA neurons with increasing chronological age of the host (e.g.: [9,10]), that proportionally increasing numbers of embryonic ventral mesencephalic (VM; the brain region containing midbrain DA neurons) neurons grafted into the parkinsonian striatum of young (3 months old), middle-aged (12 mo), and aged (22 mo) Fischer-344 (F344) rats should allow for an equal final number of surviving DA neurons. We hypothesized that if graft survival were the primary factor influencing behavioral efficacy in aged hosts, equal numbers of surviving grafted DA neurons would provide equal behavioral recovery, irrespective of the age of the graft recipient. Based on our earlier work [9], young parkinsonian rats were grafted with 200,000, middle-aged rats with 400,000, and aged rats with 900,000 embryonic VM cells. The grafts were allowed to mature and DA-responsive behavioral deficits were assessed over 11 weeks post-transplantation.

To our surprise, we found that compared to young rats, the middle-age rats showed roughly twice as many surviving grafted DA neurons, and the aged rats almost five times as many [8]. Stereological assessment further revealed that there was significantly more tyrosine hydroxylase positive (TH+) neurite outgrowth from the grafted neurons into the surrounding DA-depleted striatum in the middle-aged and aged rats. Indeed, the nearly two-fold increase in the density of neurite outgrowth in both middle-aged and aged rats compared to the young rats was a reflection of the increased numbers of surviving grafted DA neurons [8]. While neurite outgrowth was abundant in the grafted striatum of middle-aged and aged rats, when analyzed on a per cell basis, DA neurons transplanted into middle-aged and aged brains possessed significantly fewer neurites than those transplanted into the brains of young rats [8].

To examine the behavioral consequences of this grafting paradigm, parkinsonian rats were rendered dyskinetic prior to grafting by administering the anti-parkinsonian medication levodopa once-daily for 4 weeks prior to grafting to generate stable expression of levodopa-induced dyskinesias (LID) [8]. Dyskinesias, or specifically LID, are an often debilitating side-effect of DA replacement therapy that can significantly compromise quality of life for persons with PD [19]. Pretreatment with levodopa and the establishment of LID was done: 1) to emulate the status of all PD patients that have thus far received a DA neuron graft, and 2) because this complex behavioral malady can be ameliorated by DA neuron grafts in parkinsonian rats [5,11,20–22] and patients (for review [23]). Overall, the Collier et al. study [8] showed that while LID behavior was ameliorated in all DA grafted rats, the young rats with the fewest number of surviving grafted DA neurons and least amount of graft-derived neurite outgrowth, showed the fastest rate and greatest magnitude of behavioral recovery. Indeed, the middle-aged rats exhibited a pattern of delayed amelioration of LID behaviors that was eventually similar to that of young rats. However, behavioral benefit in the aged rats was delayed, and for some individual attributes of LID behaviors, completely refractory, despite survival of larger numbers of grafted cells and accompanying increased reinnervation of the host brain. Thus, despite middle-aged and aged rats having 2–5 times more surviving grafted DA neurons associated with greater neurite extension, behavioral benefit was markedly inferior with increasing age of the host.

Using confocal microscopy and dual-label immunohistochemistry (TH to label graft-derived DA neurites and synaptopodin (SP) to label dendritic spines of host MSNs) we examined the number of close appositions, presumed synaptic contacts, formed between TH+ neurites and their preferential target, the dendritic spines of host MSNs in young, middle-aged, and aged rats [8]. Despite the fact that the density of TH+ neurites was approximately 2x greater in the middle-aged and aged rats than young rats, the number of apparent synaptic contacts per grafted DA neuron (i.e.: SP contact density /TH+ cell) was found to be approximately 4–8× greater in younger rats compared to middle-aged and aged rats [8]. These data indicate that the density of contacts between TH+ neurites (graft) and dendritic spines (host), which this study found to be associated with the degree of post-graft behavioral recovery, was significantly greater for young grafted rats compared to middle-aged and aged grafted rats.

Overall these preclinical data [8] indicate that contrary to previous evidence, the aged striatum can support robust survival of grafted embryonic DA neurons. However, it further demonstrates that even when robust graft survival and neurite outgrowth are attained, currently unknown factors associated with the aged, parkinsonian striatum result in a suboptimal integration between grafted DA neurons and striatal MSNs, and inferior therapeutic efficacy.

### Additional insight from the clinic

The study in aged parkinsonian rats discussed above [8] is corroborated by a recent clinical case report in which graft-derived innervation in the striatum (i.e.: putamen) of a PD patient was extensively maintained for 24 years, despite the gradual loss of symptomatic benefits beginning 14 years post-transplantation [17]. The patient in this study underwent transplantation of VM tissue in 1989, at the age of 59 years. Dissociated tissue was deposited into posterior, middle, and anterior areas of the right putamen. The patient initially displayed dramatic bilateral clinical benefit, and levodopa was completely withdrawn after 32 months. Although low-dose levodopa was reintroduced at 74 months post-grafting, the patient maintained marked clinical benefit with reports of virtually no rigidity, minor hypokinesia, intermittent/mild resting tremor, and no on–off fluctuations for up to 12 years post-transplantation [17]. Beginning 14 years after transplantation the patient underwent a gradual loss of symptomatic benefit and by 18 years post-transplantation there was no graft-related motor improvement [17].

The patient died 24 years post-transplantation and in contrast to what might have been anticipated from the dramatic decline in graft-derived clinical benefit, postmortem analysis revealed robust survival of transplanted DA neurons in each of the three graft sites, as well as dense reinnervation of principally the dorsal putamen by the grafted cells. The immunosuppression regimen was slowly tapered and finally terminated five years post-transplantation. At autopsy there was no significant evidence of an ongoing immune response as indicated by a lack of difference between the patient and an age-matched control subject in numbers of activated microglial cells (IBA-1-positive cells, CD68-positive cells, and morphology) [17]. Thus, it is clear that transplanted DA neurons can thrive and produce significant dopaminergic reinnervation of the parkinsonian striatum even at 24 years post-transplantation, with marked clinical benefits lasting up to 14 years.

Insight from these recent reports, one in a preclinical grafting model [8] and the other in a grafted PD patient [17], corroborate the sobering facts that robust survival of grafted neurons in the aged parkinsonian striatum does not fully predict therapeutic benefit, and that even when initial benefit is achieved, aging-related changes appear to be associated with increasing graft-host dysfunction. The question of why viable, healthy appearing DA neurons that provide significant neurite projections into the surrounding striatal parenchyma fail to provide motor benefit in the aged parkinsonian striatum remains a mystery. In the remaining portion of this short communication we provide a brief discussion of how risk factors associated with the aged parkinsonian brain, namely dendritic spine loss, activation of immune/inflammatory processes, and/or alpha-synuclein (α-syn) pathology might negatively impact functional remodeling of the parkinsonian brain. Understanding such factors underlying the discordant findings of robust graft survival with limited functional benefit will doubtless be critical to the long-term success of DA terminal/cell replacement therapies for PD.

## Understanding the Impact of Individual Risk Factors

### Dendritic spine loss: Impact on graft-host reinnervation

**Figure 1:**
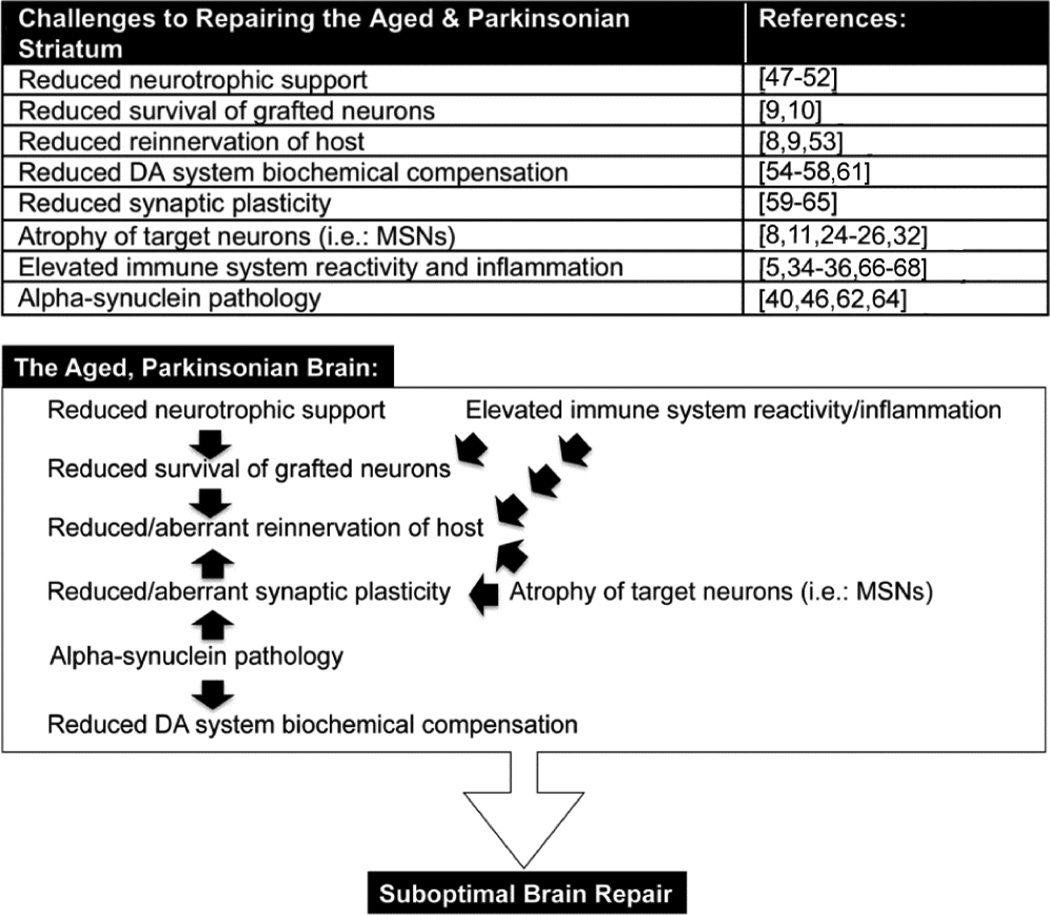
Table and Schematic Summary of Factors that Present Challenges to Repairing the Aged Parkinsonian Brain. Studies referenced in this table are found in the cited literature section of this manuscript and not intended to provide complete annotation for any particular factor presented in the table or figure; for extended details of these factors and their relationship to aging, PD, and/or grafting please see text within the manuscript

As discussed in [8], dendritic spines on MSNs in the normal striatum are critical structural elements where DA terminals predominantly synapse onto the neck of the spine and play an important modulatory role affecting afferent cortical input that synapses predominantly onto the heads of the same spines. In the parkinsonian striatum, DA depletion results in the loss of dendritic spines on MSNs (Figure 1) [11,24–26]. As a result, grafted DA neurons establish atypical synapses with the shaft of MSN dendrites more frequently than with their normal input site, the spines [5,27–31]. The functional relevance of these atypical synapses remains speculative as discussed in more detail in the following section. However, the incidence of presumed graft-derived TH+ synapses on the dendritic shaft of unlabeled host neurons is associated with the additional aberrant shift from the typical symmetrical (generally inhibitory) synapse to an asymmetrical (generally excitatory) synapse [5,31]. The incidence of these atypical asymmetric DA synapses in the grafted parkinsonian rat increases with time post-grafting and host immune response [5].

In addition to DA depletion impacting striatal spine density, it is well known in regions such as the hippocampus and neocortex that advancing age is associated with significant decrease in dendritic spines. While less is known about age-related dendritic spine changes in the striatum, a 50% loss of striatal dendritic spines has been reported in aged cats [32] and we have noted a 15% decrease of spine density in the normal, intact striatum of aged (20 mo) rats, which is further reduced with DA depletion [8]. However, the additional burden of spine density changes in the aged, parkinsonian brain on integration of grafted neurons, *per se*, remains to be fully characterized. Data from [8] indicate that the density of typical spinous synaptic contacts between graft and host neurons (i.e.: SP-TH contacts) is significantly decreased in middle-aged and aged grafted rats compared to young grafted rats. Since these contacts were correlated with the degree of LID amelioration post-grafting, this could be taken to suggest that a decrease in normal contact sites (i.e.: dendritic spines) in the aged parkinsonian striatum provides a significantly compromised environment for the establishment of normal synaptic reinnervation. This idea is supported by the finding that when striatal dendritic spine density is preserved in the parkinsonian striatum by pharmacological blockade of CaV1.3 calcium channels (the overactivity of which causes spine retraction in the DA depleted striatum [24]) there is a significant enhancement in the functional efficacy of grafted DA neurons in young parkinsonian rats [11].

Accordingly, it could be hypothesized that grafting into younger patients with less severe DA depletion would result in improved graft benefit, due at least in part to less severe loss of the critical targets (i.e.: spines) for the new, grafted DA terminals. Indeed, poor clinical outcome after DA neuron transplantation in PD patients is associated with more substantive DA denervation in areas outside the grafts [1] and with patients of more advanced age [2]. However, the question remains that even if graft therapy is aimed at younger patients with less severe disease, can graft benefit be maintained in the face of progressive pathology associated with PD? For example, as introduced above, within the striatum (putamen) of PD patients, loss of MSN dendritic spines will be an event that potentially occurs early in the disease and progresses over time with continuing loss of striatal DA. This pathology culminates in markedly truncated dendrites that ultimately contain only sparse numbers of spines and irregular, bulbous swellings [26,33]. These significant morphologic changes are thought to underlie the declining efficacy of chronic levodopa replacement therapy in advanced PD [26,33]. It is reasonable to suggest that if these significant morphological changes continue to occur in the grafted striatum, they would readily contribute to deafferentation of the inputs that might have been established early after grafting. Gradual deafferentation combined with disease progression could be hypothesized to contribute to loss of graft-derived benefit [17].

### Host immune response

To date, there is no standard approach to immune suppression in neural transplantation. However, the potential importance of an interaction between immune/inflammatory processes and the function of grafted neurons has been suggested by findings from one clinical trial [2]. In this trial, immunosuppressant drugs were administered to patients receiving grafts prior to, and for six months following transplant surgery. In some patients, initial therapeutic benefit was gradually lost and graft-related complications (graft-induced dyskinesia: GID) emerged along a time course that corresponded to discontinuation of immune suppression. Post-mortem analysis of two patients from this trial revealed pronounced activation of microglial cells surrounding grafted neurons. The potential contribution of immune/inflammatory processes to graft dysfunction is likely exaggerated in the aged brain. Indeed, a prominent feature of normal brain aging is neuroinflammation, which has been linked to aging-related synaptic dysfunction (for review [8]). In addition to normal aging, DA-depletion and cell transplantation are commonly associated with immune activation and neuroinflammation (Figure 1) [5,34–36]. Although Li et al. [17] reported no differences in microglial activation between the grafted PD patient and an age-matched control individual, this snapshot in time does not rule out the presence of immune/inflammatory activation during some period in the history of the transplant. Furthermore, while overt rejection of grafted neurons is not seen, the likelihood of synaptic dysregulation secondary to age-related neuroflammation cannot, and should not be ruled out.

The importance of continued consideration of immune function/dysfunction in DA neuron grafting in PD is supported by previous work showing that in grafted parkinsonian rats, activation of the immune system can have profound effects on the pattern and ultrastructure of graft-derived synaptic contacts [5]. Specifically, these data unequivocally demonstrate that the atypical TH+ axo-dendritic (as opposed to normal TH+ axo-spinous) synapses are cytoarchitectural features that are exacerbated by immune activation and that these aberrant synapses in a preclinical model correlate statistically with the deleterious side-effect known as GID. While the mechanism(s) underlying GID remain uncertain, and the patient in [17] experienced very mild off-time dyskinesias (i.e.: GID) on the side contralateral to the graft, the impact of immune activation/neuroinflammation on DA neuron graft therapy warrants continued investigation.

### Alpha synuclein and graft dysfunction

PD is one of several neurodegenerative diseases that are classified as synucleinopathies. This particular type of disease is characterized by the abnormal accumulation of filamentous, insoluble forms of the protein α-syn. Physiological α-syn, however, is soluble and typically localized to presynaptic terminals. Its function in the synapse is not well understood, though evidence suggests that α-syn is involved in synaptic maintenance and function (e.g.:[37,38]).

While α-syn is found in widespread regions of the vertebrate brain (for review [39]), intracytoplasmic α-syn inclusions known as Lewy bodies (LBs) and Lewy neurites (LNs) within nigral DA neurons are the histological hallmark of PD pathology. In PD, LBs are usually found in the cell body of surviving nigral DA neurons, though they also are found in other catecholaminergic neurons of the brainstem [37]. They are composed of filamentous α-syn and typically appear as a dense, spherical core of protein with a surrounding ring of lighter coloration. Similarly, LNs involve insoluble α-syn inclusions that localize to the axon, particularly in early stages of synucleinopathies including PD [40]. The accumulation of α-syn into LBs and LNs is traditionally believed to be a toxic gain-of-function event leading to death of DA neurons, though more recent evidence suggests a possible loss-of-function may instead occur related to displacement of physiological α-syn by misfolding and aggregation (e.g., [41]). This issue is a current topic of debate.

Recently, the discovery of LBs in grafted DA neurons prompted a new line of thought regarding the intercellular spread of α-syn as a component of PD pathology. In 2008, two groups reported LBs and LB-like inclusions in grafts of VM DA neurons from patients grafted more than a decade previously [13,15]. More recent autopsy reports, including the case report discussed above [17], have corroborated these findings [12,14,42,43]. The spread of α-syn from the host brain to grafted neurons also has been verified in rodent models of PD [44,45].

The discovery of LBs in grafted DA neurons appeared to confirm the long-feared notion that PD pathology could spread from host to graft, indicating that even young, healthy, transplanted DA neurons have limited time in the parkinsonian brain. Recall that the grafted PD patient described by Li et al. [17] experienced a gradual decline of graft-derived benefit. Despite the overall appearance of transplanted neurons appearing phenotypically normal, postmortem histological analysis revealed LBs in 11–12% of the grafted neurons. Such findings beg the vital question of whether α-syn accumulation in grafted DA neurons may somehow perpetuate gradual loss of graft efficacy observed in patients whose grafts contain α-syn pathology [13,17]. However, since the incidence of LBs is generally 1–5% of total grafted neurons, with the highest incidence being 11–12% in a 24 year-old graft [17], the likelihood of this pathology contributing to demise of clinical benefit has been considered to be improbable.

On the other hand, the presence of LBs and LNs in grafted neurons are downstream markers of earlier pathology thought to be initiated as a consequence of misfolding of α-syn protein and the seeding of small, insoluble aggregates known to form first in axons, followed by the formation of somatodendritic α-syn inclusions and aggregates that lead to the formation of LBs [40,41]. Early events including the formation of LNs have been demonstrated to result in selective alterations in axonal transport and defects in neuronal synchronization early in the disease process [40]. In DA neurons, the absence or aggregation of α-syn also is associated with alterations in synaptic vesicle clustering and a significant decrease in basal and depolarization-dependent DA release [46]. Thus, synaptic dysfunction associated with evolving α-syn pathology may be more widespread in grafted neurons and is not readily detected in post-mortem examination (Figure 1).

## Conclusions

The aging brain provides a challenging environment for recapitulation of nigrostriatal function by grafted DA neurons in PD and its models. Aging-associated diminished trophic factor support, blunted biochemical compensatory mechanisms, activation of immune/inflammatory processes and continued evolution of other factors contributing to pathology can conspire to limit therapeutic benefit (Figure 1). The factors at work clearly extend beyond adequate survival of grafted neurons, as cell survival was impressive in both the preclinical study and clinical case report discussed here. While the challenges presented by the environment of the aging brain may not be insurmountable, at present they are incompletely understood.
